# Editorial Retirement of Dr. Mundé

**Published:** 1892-01

**Authors:** 


### EDITORIAL RETIREMENT OF DR. MUNDÉ.

Dr. Paul F. Mundé announced in the December number of the *American Journal of Obstetrics* that at the close of the year he would retire from the editorial management of that journal.

For eighteen years Dr. Mundé has successfully conducted the first journal in this country to be devoted to the consideration of a special department of medicine. During this period the life of Dr. Mundé has been so completely interwoven with that of the *American Journal of Obstetrics*, that it is difficult to think of the one without indistinctively being minded of the other. To say that this long editorial career has been a complete success is only to repeat what everybody in the profession already knows.

At the annual dinner of the American Association of Obstetricians and Gynecologists given at the Academy of Medicine, in the City of New York, on the evening of September 18, 1891, Dr. Mundé was invited to respond to the sentiment *The American Journal of Obstetrics.* In the course of his timely and eloquent little speech, he referred to the fact that this change was about to occur, and he further remarked, that in his whole editorial career, this was the first occasion when the journal, with which he had been so indissolubly connected for eighteen years, had been noticed in a public manner, or spoken of in a conspicuous way at a banquet. He seemed as much touched by the references to his journal as those present were by his speech, and especially by the fact that it foreshadowed his retirement from the editoral management thereof.

He leaves the journal so thoroughly established in the hearts of the profession, that it need have no fear of being supplanted by any other periodical. There may be, and no doubt is, a field for other good journals, but the *American Journal of Obstetrics* will live as long as the science which it expounds is recognized as a distinct specialty.

Dr. Mundé commits the journal to an able successor, Dr. Brooks H. Wells, who has been his assistant in its conduct for several years, and who has won the respect and affection of every one of the contributors and patrons of the journal with whom he has come in contact.

We regret that Dr. Mundé must retire, but since this must be, we congratulate the publishers upon securing the services of so able and polished a successor. In bidding adieu to Dr. Mundé, we hail Dr. Wells.

